# Using Mosses as Bioindicators of Potentially Toxic Element Contamination in Ecologically Valuable Areas Located in the Vicinity of a Road: A Case Study

**DOI:** 10.3390/ijerph16203963

**Published:** 2019-10-17

**Authors:** Maja Radziemska, Zbigniew Mazur, Agnieszka Bes, Grzegorz Majewski, Zygmunt M. Gusiatin, Martin Brtnicky

**Affiliations:** 1 Faculty of Civil and Environmental Engineering, Warsaw University of Life Sciences—SGGW, Nowoursynowska 159, 02-776 Warsaw, Poland; grzegorz_majewski@sggw.pl; 2 Faculty of Environmental Management and Agriculture, University of Warmia and Mazury in Olsztyn, Pl. Lodzki 4, 10-727 Olsztyn, Polandagnieszka.bes@uwm.edu.pl (A.B.); 3 Faculty of Environmental Sciences, University of Warmia and Mazury in Olsztyn, Słoneczna St. 45G, 10-719 Olsztyn, Poland; mariusz.gusiatin@uwm.edu.pl; 4 Department of Geology and Pedology, Faculty of Forestry and Wood Technology, Mendel University in Brno, Zemedelska 1/1665, 613 00 Brno, Czech Republic; 5 Central European Institute of Technology, Brno University of Technology, Purkynova 656/123, 612 00 Brno, Czech Republic

**Keywords:** bioindicators, road transportation, potentially toxic elements, *Pleurozium schreberi*

## Abstract

This study analyzed the impact of road transportation on the concentration of Zn, Ni, Pb, Co, and Cd in moss (*Pleurozium schreberi*). The study was carried out over five years near a national road running from the north to the east (Poland) in the area of Natura 2000 sites. Samples were collected at three significantly different locations: (1) near a sharp bend, (2) near a straight section of the road in a woodless area, and (3) in a slightly wooded area. At each location, moss samples were collected from sites situated 2, 4, 6, 8, 10, 12, and 14 m from the road edge. The highest Zn and Cd contents in the moss were recorded 6 m from the road edge near a sharp bend (where vehicles brake sharply and accelerate suddenly). At the same location, at a distance of 2 m, the highest Pb concentration was noted, and at a distance of 4 m from the road, the highest Ni concentration was noted. The Co concentration in the moss was the highest near the woodless straight section at a distance of 2 and 12 m from the road. The concentrations of Zn, Pb, Ni, Co (only at the woodless location), and Cd (at all locations) were significantly and negatively correlated with distance from the road.

## 1. Introduction

Potentially toxic elements are a group of harmful chemical compounds accumulated in the environment as a result of anthropogenic factors [1,2,3,4]. Their excessive concentration has an adverse effect both on human health and on particular components of the natural environment [5]. Road transportation, alongside industry and agriculture, is the main anthropogenic source of environmental pollution with potentially toxic elements [6,7,8]. The transportation impact on the environment is multidirectional and primarily concerns air, water, and soil conditions; biodiversity disturbances; area development; noise; and vibration hazards. The development of the automotive industry in recent decades has contributed to an increase in the emissions of toxic exhaust fume components, including potentially toxic elements. Car exhaust fumes as well as rubber microparticles from tires and brake linings generate characteristic dustiness at locations with a high traffic volume [9]. Cars are responsible for 10–25% of dust emissions, and their contribution to contamination is even greater due to the phenomenon of secondary dusting caused by car wheels [10]. Road transportation is among the main sources of air pollutant emissions that pose a threat to the natural environment as well as to human health and lives. Fuel combustion, operation fluid leakage, and the wear of brake pads, clutch plates, road surfaces, and tires lead to the emission of gases, liquids, and dusts containing potentially toxic elements such as Pb, Cd, Ni, Zn, Cu, and Co [11,12]. These emissions can be transported over various distances depending on the geographical area and meteorological factors, including fog, wind, and rain [13]. The spatial distribution of contamination with toxic elements caused by road transportation also depends on the organization of road traffic and road infrastructure. For example, in Poland, road infrastructure occupies approximately 3% of the country’s land area, but approximately 50% of Poland’s land area is within the range of direct impacts from transportation pollution [14].

Biomonitoring can be applied to assess the effect of road pollutants on particular components of the natural environment. Research carried out in this field enables a determination of the type, scale, and rate of adverse changes and the preparation of a forecast for expected environmental pollution. Bioindicators are used for both qualitative and quantitative assessments of environmental conditions and the degree of environmental transformation (to indicate measurable morphological, anatomical, and physiological changes). Mosses are recognized as one of the main bioindicators and biomonitors of air pollution, including those originating from road transportation. In Poland, a species frequently used for air quality control is the moss *Pleurozium schreberi* (Brid.) Mitt. Mosses are commonly found worldwide and have a large surface area in relation to their weight [15]. Mosses have no roots and take up most of the nutrients from (wet and dry) precipitation. They are capable of accumulating potentially toxic elements over a long period of time up to very high concentrations [16] that are determined by the moss species [17]. The concentration of potentially toxic elements in exposed moss samples results from two basic processes, i.e., dust retention on the plant surface and the take-up of substances in their ionic form. With regard to dry deposition, potentially toxic elements remain practically inactive and are absorbed mechanically. However, when high concentrations of acidifying gases and precipitation occur at the same time, the bioaccumulation of more readily soluble ionic potentially toxic elements forms increases. *P. schreberi* mosses have also been rather widely used in biomonitoring research worldwide: in Albania [18], China [19], Finland [20], North America [21], Sweden [15], the Ural region [22], Canada [23], and Poland [24,25,26]. However, long-term monitoring studies with mosses are not frequently performed. It is important to assess how transportation emissions affect environmental pollution along a road depending on different landforms, afforestation densities, and limits of vehicle speed.

The aim of this study was to assess the effect of road transportation on potentially toxic element (Zn, Pb, Ni, Cd, and Co) contents in samples of the moss *P. schreberi*, which were collected over five years of research along a national road in northeastern Poland located within an ecologically valuable area (Natura 2000). The main objectives of this study were (1) to determine the spatial distribution of toxic elements in moss along a national road, and (2) to analyze the effect of the distance from transportation emissions at a specific location near the road on the content of potentially toxic elements in moss.

## 2. Materials and Methods

### 2.1. Sampling Strategy

In this study, selected potentially toxic element contents were analyzed in moss (*P. Schreberi*) samples collected at three locations situated along National Road No. 53 between Olsztyn and Szczytno (northeastern Poland) near Lake Kosno, where the road marks the northern boundary of the Lake Kosno Landscape Reserve. This enclave is a part of the Napiwodzko–Ramucka Refuge and is a site of community importance (the Natura 2000 program). The road is characterized by a high average traffic volume of approximately 5000 cars per day, of which 80% are passenger cars.

**Figure 1:**
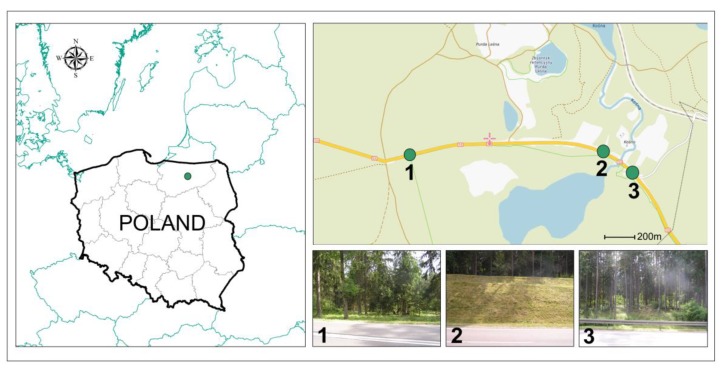
The location of sample collection sites.

Sample collection and storage was carried out in line with European moss network guidelines [22]. Moss samples were collected over five years (2014–2018) in July, each time at three sampling points (Figure 1). Table 1 presents the meteorological conditions in the sampling points of the region in July. The locations differed in landform, afforestation density, and limit of vehicle speed. Location 1 (53°40′12.3″ N; 20°42′38.4″ E) was situated near a sharp bend (a thin pine forest). The travel speed is limited here to 40 km·h^−1^ (at other sampling points to 90 km·h^−1^). The terrain is flat. Up to 19 m from the road, there is a rare spruce forest that passes further into a dense pine and spruce forest. Location 2 (53°40′15.9″ N; 20°41′51.0″ E) was situated close to a straight road section and is characterized by a high scarp (40 m long) near the road (the slope toward the road was 20°). The terrain is woodless and covered by grass. The thick forest is 50 m from the edge of the road. Location 3 (53°40′6.7″ N; 20°42′18.3″ E) lay at the site of a forest clearing with a surrounding thin pine forest. The terrain gently slopes off the road by 3°. Similarly to location 2, research points were located close to a straight road section (high speed of vehicles).

In this area, winds from the southwestern and western directions prevail. At each location, moss samples were collected from sites situated 2, 4, 6, 8, 10, 12, and 14 m from the road edge. Each sample consisted of 10 subsamples (0.5 g for each one) taken over an open space area of 1 × 0.1 m (collected parallel to the road) and mixed to make a single, representative sample. Moss samples were collected at locations at least seven meters away from the nearest tree trunk to avoid direct exposure to throughfall. In total, 1050 moss samples were collected, of which 105 representative samples were prepared. Samples were stored and transported to the laboratory in tightly closed paper bags.

### 2.2. Plant Material Analyses

Green parts of mosses were dried at room temperature. The plant samples were homogenized to a fine powder in a Retsch-type ZM300 (Hann, Germany) laboratory mill. Microwave digestion (MARS6, CEM Corporation, Matthews, USA) in nitric acid (HNO_3_ 65%, suprapure) was used for total digestion of the moss samples. The total contents of Zn, Ni, Pb, Co, and Cd were determined using the flame atomic absorption spectroscopy (FAAS) method with a SpectrAA 240FS spectrometer (VARIAN, Mulgrave, Australia). The detection limits were 0.09, 0.26, 0.48, 0.24, and 0.20 μg·g^−1^ for Cd, Co, Ni, Pb, and Zn, respectively. Ultrapure water (Merck, Darmstadt, Germany) with 0.055 µS·cm^−1^ resistivity was used for preparing the working standard solutions and sample dilutions. All glass and polyethylene flaskware had been previously treated in 5 mol·L^−1^ HNO_3_ for 24 h and then rinsed with ultrapure water. Calibration of the spectrometer was performed with a standard solution from Merck (Darmstadt, Germany). The accuracy of the potentially toxic element analysis using FAAS was checked through an analysis of the reference materials (BCR-482, BCR-414). The concentrations of Cd, Ni, Pb, and Zn that were recovered were satisfactory, ranging from 94% to 109%.

### 2.3. Statistical Analysis

The study results were processed statistically by calculating the average values of potentially toxic elements and standard deviations. Coefficients for Pearson’s simple correlation between the determined potentially toxic element contents of the moss and the distance from the road edge were calculated as well. In order to determine the significance of differences in the concentration of potentially toxic elements between the research points and distances from the road, Tukey’s test was applied (*p* < 0.01). An analysis of variance (ANOVA) was used to compare the effects of sampling location (factor 1) and distance from the road (factor 2) on the content of potentially toxic elements in moss, and the interaction between these factors was also included. The response variable in ANOVA was the concentration of potentially toxic elements, calculated as the mean of sampling years. The statistical analysis was conducted using STATISTICA 13.3 software [27].

## 3. Results and Discussion

Road transportation is among the major factors of global economic development as well as one of the main sources of air pollutant emissions that pose a threat to the natural environment and human health [28,29]. The most vulnerable to automotive pollution are areas found in the vicinity of roads, particularly in cities [30]. The greatest threats to the environment surrounding traffic routes are potentially toxic elements emitted primarily by road transportation [31,32].

**Figure 2:**
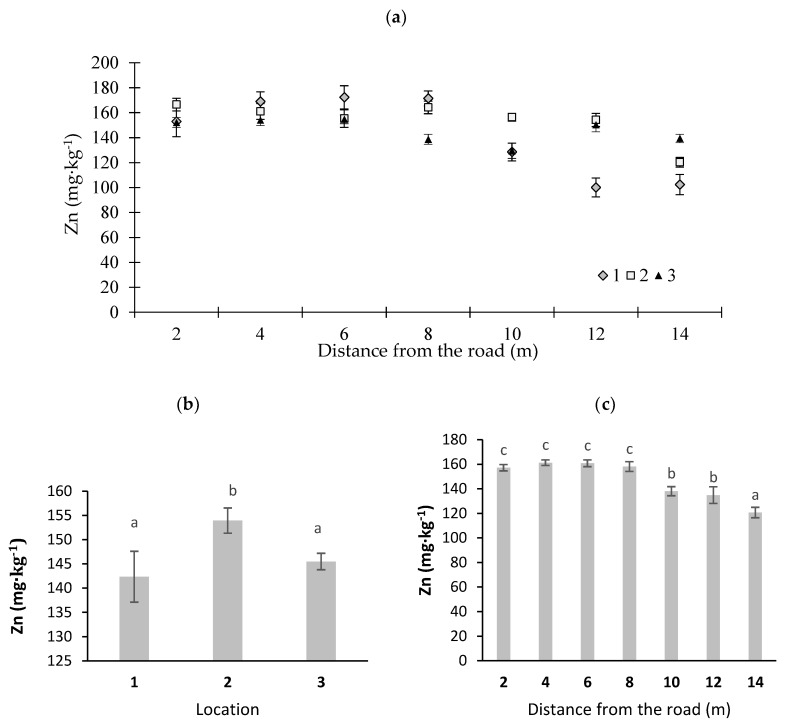
Zinc concentrations in moss (*Pleurozium schreberi*): (**a**) distance from the road at sampling location, (**b**) average Zn content at different locations, and (**c**) Zn content at different distances from the road (average values from three locations) (*n* = 5, ±standard deviation). Different letters above bars representing lead concentrations for different locations or for different distances from the road indicate that they differed significantly (Tukey’s test, *p* < 0.01).

**Figure 3:**
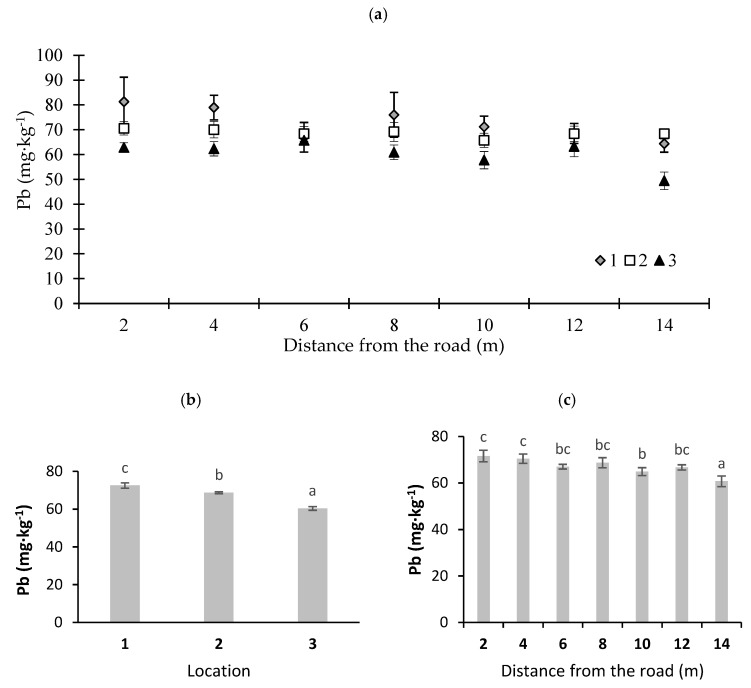
Lead concentrations in moss (*P. schreberi*): (**a**) distance from the road at sampling location, (**b**) average Pb content at different locations, and (**c**) Pb content at different distances from the road (average values from three locations) (*n* = 5, ±standard deviation). Different letters above bars representing lead concentrations for different locations or for different distances from the road indicate that they differed significantly (Tukey’s test, *p* < 0.01).

**Figure 4:**
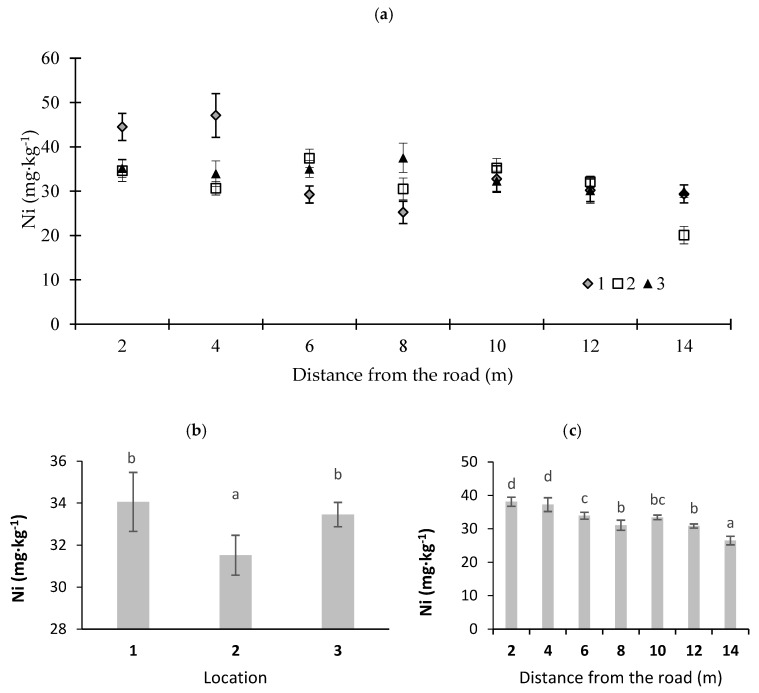
Nickel concentrations in moss (*P. schreberi*): (**a**) distance from the road at sampling location, (**b**) average Ni content at different locations, and (**c**) Ni content at different distances from the road (average values from three locations) (*n* = 5, ±standard deviation). Different letters above bars representing lead concentrations for different locations or for different distances from the road indicate that they differed significantly (Tukey’s test, *p* < 0.01).

**Figure 5:**
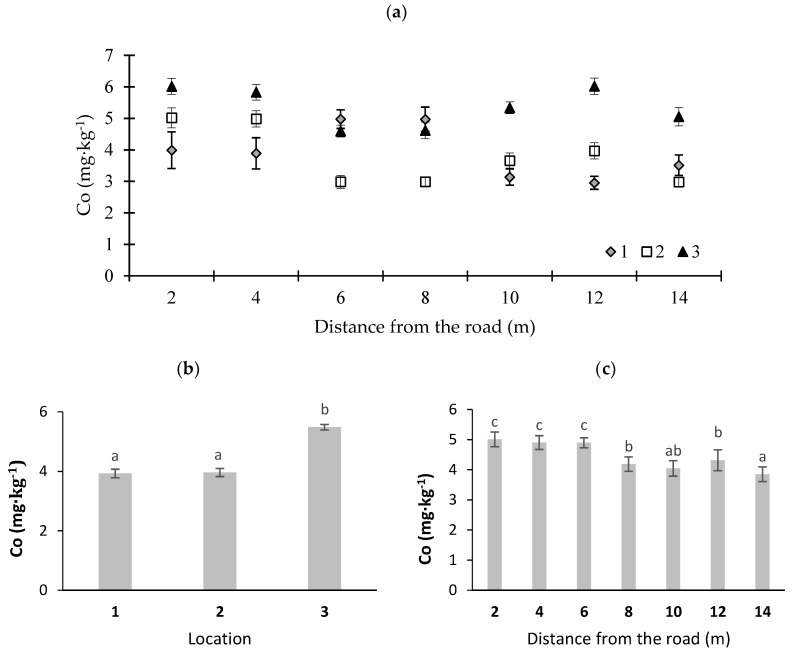
Cobalt concentrations in moss (*P. schreberi*): (**a**) distance from the road at sampling location, (**b**) average Co content at different locations, and (**c**) Co content at different distances from the road (average values from three locations) (*n* = 5, ±standard deviation). Different letters above bars representing lead concentrations for different locations or for different distances from the road indicate that they differed significantly (Tukey’s test, *p* < 0.01).

**Figure 6:**
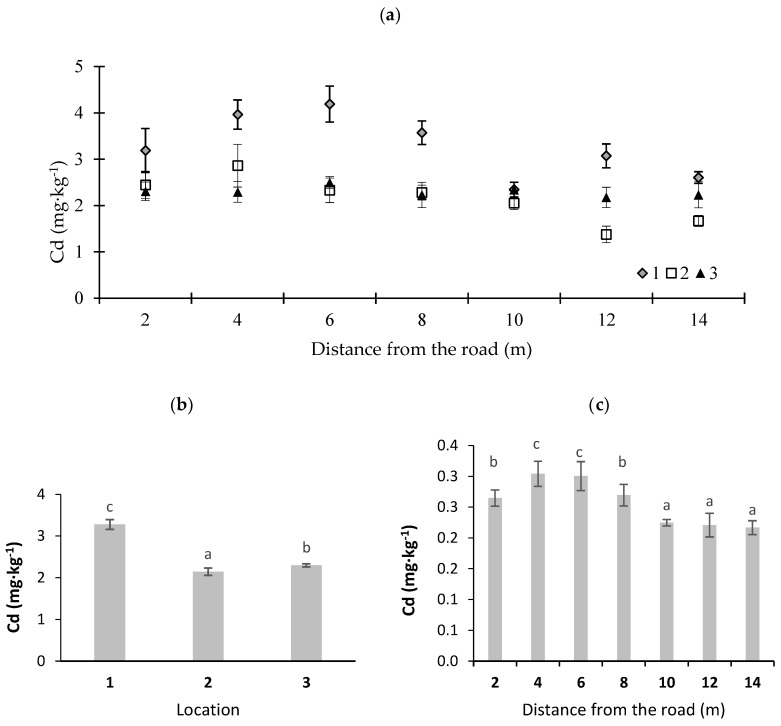
Cadmium concentrations in moss (*P. schreberi*): (**a**) distance from the road at sampling location, (**b**) average Cd content at different locations, and (**c**) Cd content at different distances from the road (average values from three locations) (*n* = 5, ±standard deviation). Different letters above bars representing lead concentrations for different locations or for different distances from the road indicate that they differed significantly (Tukey’s test, *p* < 0.01).

In Figure 2, Figure 3, Figure 4, Figure 5 and Figure 6, potentially toxic elements in moss are presented. According to Johansson et al. [33], an increased concentration of Zn in the vicinity of roads is due to fuel and oil combustion in worn engines and the emissions of dust from brake pads. Councell et al. [34] and Sjodin et al. [35] stated that Zn in road dust originates from tires and asphalt becoming worn out. Cowden and Aherne [36] found that Zn did not correlate with their modeled deposition data, which may insinuate other influences that dictate tissue concentrations for that potentially toxic element. In this study, the highest Zn concentration was noted in the moss collected at sampling point 1 (the sharp bend) (Figure 2). This may have been due to the fact that cars at this sampling point often change speed, which leads to the abrasion of vehicle wear parts and an increase in emissions of this element. With distance from the road, Zn content was decreased (at *p* < 0.01) (Figure 2c). For example, at location 1, Zn content was 172.1 mg·kg^−1^ at a distance of 6 m, and it decreased to 100.0 mg·kg^−1^ dry mass at a distance of 12 m from the road edge (Figure 2a). Similar results were noted by Korzeniowska and Panek [37], who studied mosses near roads in southern Poland. The average Zn content in moss at different locations varied from 142 to 154 mg·kg^−1^ dry matter (Figure 2b) and significantly exceeded the average Zn concentration for Poland, which amounts to 53 mg·kg^−1^ dry matter [38].

Among the studied elements, the greatest threat to the environment is Pb pollution, and road transportation is among the major sources of Pb emissions [39]. Nowadays, due to the widespread use of unleaded petrol, this threat has significantly decreased. However, according to Winther and Slentø [40] and Johansson et al. [33], a low Pb concentration in exhaust fumes is still observed. Location and distance from the road affected the Pb content in moss. The average content of this element in the moss ranged from 60 mg·kg^−1^ at the point farthest from the road at location 3 to 72 mg·kg^−1^ dry matter at location 1 at the point situated closest to the road (Figure 3b), and it was many times higher than the average concentration for Poland (17.3 mg·kg^−1^) [38]. With distance from the road at different locations, Pb content in moss also decreased (Figure 3a,c). The results obtained by Bakirdere and Yaman [41], which described Pb content in plants, indicate that despite the phase-out of Pb-containing petrol, it is still the greatest source of this potentially toxic element in the environment. Blagnytė and Paliulis [42], who examined the concentration of Pb in *Pylaisia polyantha* (Hedw.) Schimp in the vicinity of a road with a high road traffic volume in Vilnius, obtained results similar to the present study.

Certain amounts of Ni are found in fuels, engine oils, and steel [40]. The Ni content in moss showed some fluctuations. The average Ni content in the moss at the analyzed locations ranged from 26.5 mg·kg^−1^ at a distance of 14 m from the road edge to 38.1 mg·kg^−1^ at a distance of 2 m from the road edge (Figure 4c). The highest concentration of Ni was found at the sampling point near a sharp bend. The median value determined for samples collected in the Karkonosze region in 2014–2016 was 0.5 mg Ni·kg^−1^ [25]. The highest concentration of this element was noted at location 1 at a distance of 4 m from the road (47.1 mg·kg^−1^). Grodzinska et al. [43] determined the average contents of this trace element in Poland to be a value ranging from 1.4 to 2.3 mg·kg^−1^ dry matter. A relationship between the Ni concentration in moss and the distance from a busy expressway or a busy city road was also found by Korzeniowska and Panek [37] and Blagnytė and Paliulis [42].

Among the analyzed contents of potentially toxic elements in moss, the greatest fluctuations were observed for Co (Figure 5). The highest Co contents in moss (6.0 mg·kg^−1^ dry matter) were noted at location 3 at a distance of 2 and 12 m from the road edge (Figure 5). The average Co contents in moss from locations 1 and 2 were comparable (*p* < 0.01) (Figure 5b). Despite fluctuations in Co contents, with an increase in distance from the road, Co content decreased (Figure 5c). A study conducted by Grodzinska et al. [43] demonstrated that the average Co content in moss in Poland is 0.3 mg·kg^−1^. The maximum contents of this element reached a value of 2.0 mg·kg^−1^ dry matter in unpolluted areas of northeastern Poland. The relationship between Co concentrations in moss and road traffic was demonstrated by Zechmeister et al. [44].

Cd is a very toxic element, and its presence near roads is attributed to dust from the combustion of petrol, to brake linings, and to the rubber used for tire production [37]. Sources of Cd in the vicinity of roads are the combustion of engine oils and the wear of tires and asphalt [37]. In the present study, Cd contents in moss were evidently the highest at location 1 (Figure 6). This is a place where cars brake sharply and accelerate suddenly. At that sampling location, at a distance of 6 m from the road, the Cd content in the moss was high (4.2 mg·kg^−1^). On the other hand, the lowest concentration of this element (1.4 mg·kg^−1^ dry matter) was noted at location 2 at a distance of 12 m from the road (Figure 6a). The highest concentration of the element was found in the sampling location situated near a sharp bend. This indicates that trees around the road were a natural barrier and helped to keep potentially toxic elements from being spread to the neighborhood. At a distance of 10 m and more from the road, the Cd content was the lowest (Figure 6c). Korzeniowska and Panek [37] noted that the highest Cd content (1.4–2.8 mg·kg^−1^) in moss was at a distance of 50 m from the DK7 and DK47 roads in southern Poland. Grodzinska et al. [43] reported that the Cd content in moss may reach high values exceeding 16 mg·kg^−1^ dry matter in industrial regions in Poland (Silesian–Krakow Region).

Table 2 presents the correlation coefficients between the contents of potentially toxic elements in moss. Strong positive correlations were noted between all analyzed potentially toxic elements in *P. schreberi.* The highest correlation (*r* = 0.92) was observed between Zn and Cd and the lowest between Ni and Cd and between Pb and Cd (*r* = 0.65 and *r* = 0.68, respectively). Similar correlations between Zn, Ni, Pb, and Co in the road dust were shown in a study by Johansson et al. [33]. A very high correlation (*r* = 0.96) between Pb and Cd (emitted by road traffic) was demonstrated by Mazur et al. [45]. Sudip et al. [46] found that in road dust studied over four months, the correlation coefficients (*r*) between Zn, Cd, Ni, and Pb ranged from 0.43 to 0.74 and were determined by distance from the curb.

In this study, there was a very strong, negative correlation between content of potentially toxic elements in *P. schreberi* and the distance from the emissions source at a given sampling location. This means that the content of potentially toxic elements decreased with distance from the road at different locations. In addition, an analysis of variances showed a statistically significant influence of the analyzed factors (location and distance from the road) and their interactions on the concentration of potentially toxic elements in *P. schreberi* (Table 3).

## 4. Conclusions

The results from the five-year study indicate that road transportation is a source of roadside area pollution through potentially toxic elements. The contents of analyzed elements in the moss (*P. schreberi*) were a few or several times greater than the average contents noted in Poland. The highest Zn and Cd contents in the moss at the analyzed road sections were noted at a distance of 6 m from the road edge near a sharp bend where vehicles brake sharply and accelerate suddenly. At the same location, at a distance of 2 m, the highest Pb concentration was noted, and at a distance of 4 m from the road, the highest Ni concentration was noted. The Co concentration in the moss was the highest near a woodless straight Section 2 and 12 m from the road. The concentrations of Zn, Pb, Ni, Co, and Cd were significantly negatively correlated with distance from the road. Moreover, strong correlations were noted between the potentially toxic element contents analyzed in the moss, which indicates their common origin.

## Figures and Tables

**Table 1:** Meteorological conditions at the sampling points of the region in July.

		2014	2015	2016	2017	2018
***T***	°C	21.0	17.8	18.3	17.1	19.5
***T*max**	°C	26.7	23.6	23.3	21.8	19.5
***T*min**	°C	15.3	11.5	13.9	12.2	14.5
***RH***	%	64.5	68.4	78.1	75.6	75.1
***WS***	km·h^−1^	9.6	10.0	9.1	9.9	11.4
***P***	mm	30.5	84.4	114.5	121.7	128.3
***AP***	hPa	1014.6	1013.6	1014.5	1011.4	1012.9

*T*: air Temperature; *RH*: relative Humidity; *WS*: wind Speed; *AP*: atmospheric Pressure; *P*: precipitation.

**Table 2:** Pearson’s correlation coefficients (*r*) between contents of potentially toxic elements in moss from five-year monitoring research (significant at ** *p* < 0.01 and * *p* < 0.05).

	Distance	Zn	Pb	Ni	Co
Zn	−0.89 **				
Pb	−0.89 **	0.86 *			
Ni	−0.93 **	0.77 *	0.86 *		
Co	−0.90 **	0.81 *	0.81 *	0.86 *	
Cd	−0.80 *	0.92 **	0.68	0.65	0.80 *

**Table 3:** Analysis of variances (*F*-test) of the contents of potentially toxic elements in moss from five-year monitoring research (significant at ** *p* < 0.01).

Source of Variation	Degrees of Freedom	*F*-Values and Significant Levels of Fixed Effects				
		Zn	Co	Pb	Ni	Cd
**Location (A)**	2	31.3 **	317 **	68.3 **	9.56 **	192 **
Distance from the road (B)	6	95.9 **	38.0 **	10.0 **	37.1 **	30.1 **
A x B	12	36.6 **	28.5 **	4.11 **	23.8 **	11.0 **
Error	84					
